# Spatial epidemiology in zoonotic parasitic diseases: insights gained at the 1^st ^International Symposium on Geospatial Health in Lijiang, China, 2007

**DOI:** 10.1186/1756-3305-2-10

**Published:** 2009-02-04

**Authors:** Xiao-Nong Zhou, Shan Lv, Guo-Jing Yang, Thomas K Kristensen, N Robert Bergquist, Jürg Utzinger, John B Malone

**Affiliations:** 1 National Institute of Parasitic Diseases, Chinese Center for Disease Control and Prevention, Shanghai, PR China; 2 Department of Public Health and Epidemiology, Swiss Tropical Institute, Basel, Switzerland; 3 Jiangsu Institute of Parasitic Diseases, Wuxi, PR China; 4 DBL – Institute for Health Research and Development, University of Copenhagen, Denmark; 5 Ingerod, Brastad, Sweden; 6 Department of Pathobiological Sciences, School of Veterinary Medicine, Skip Bertman Drive, Louisiana State University, Baton Rouge, LA, USA

## Abstract

The 1^st ^International Symposium on Geospatial Health was convened in Lijiang, Yunnan province, People's Republic of China from 8 to 9 September, 2007. The objective was to review progress made with the application of spatial techniques on zoonotic parasitic diseases, particularly in Southeast Asia. The symposium featured 71 presentations covering soil-transmitted and water-borne helminth infections, as well as arthropod-borne diseases such as leishmaniasis, malaria and lymphatic filariasis. The work made public at this occasion is briefly summarized here to highlight the advances made and to put forth research priorities in this area. Approaches such as geographical information systems (GIS), global positioning systems (GPS) and remote sensing (RS), including spatial statistics, web-based GIS and map visualization of field investigations, figured prominently in the presentation.

## Background

The goal of the '1^st ^International Symposium on Geospatial Health', convened in Lijiang, Yunnan province, People's Republic of China from 8 to 9 September, 2007, was to review advances made in the control of zoonotic parasitic diseases through the use of geospatial tools. The symposium, organized by the Global Network for Geospatial Health  and supported by the Ministry of Health (MoH) of China, the World Health Organization (WHO), and the UNICEF/UNDP/World Bank/WHO Special Programme for Research and Training in Tropical Diseases (TDR) [1], was held in conjunction with the '7^th ^Annual Meeting of the Regional Network for Asian Schistosomiasis and Other Zoonotic Helminthiases' (RNAS^+^; ) [2]. It attracted more than 150 participants from 19 countries/regions and international organizations and the 71 presentations, including 10 plenary sessions, dealt with intervention strategies, risk profiling, spatio-temporal modeling of parasitic disease transmission, biological investigations to further our understanding of the interaction between vectors and/or intermediate hosts with the definitive human host, as well as database management and sharing of data. Geostatistical approaches and time series analyses have been employed in schistosomiasis control in China, including random-effect modeling, transmission dynamics and Bayesian geostatistics.

The symposium was essentially an initiative intended to encourage local and international scientists to share data and geospatial health applications in compatible format with special emphasis on the region represented by the site of the annual RNAS^+ ^meeting. It had the form of an open forum where the information from different regions and diseases was freely exchanged. Results from simple cross-sectional surveys as well as advanced modeling, such as that based on random-effects, spatio-temporal studies or transmission dynamics, were presented along with Bayesian statistics and multidisciplinary theory. The overarching theme was 'Towards a Regional Information System for Control of Parasitic Diseases in the Greater Mekong Sub-Region'.

In the remote past, doctors could only observe the progress of, say the Black Death, but were powerless to interfere due to inadequate understanding of the cause, while we now have an essentially full understanding of infectious agents and their interrelation with humans, vectors and intermediate hosts [3,4]. Moreover, technical progress in recent decades has provided the necessary instruments to study these interrelationships in great detail using geographical information systems (GIS) and satellite-based remote sensing (RS). This new approach to the epidemiology has come into its own during the last few years as it has become increasingly clear that, from the geological point of view, we live in a period of rapid environmental transformation [5-7]. Already, areas have been identified where new incursions of diseases can be expected, e.g., schistosomiasis in northern China [8], malaria at higher altitudes in Africa [9], and zoonotic diseases in southern Europe [10]. GIS and RS provide the early-warning tools needed to permit preemptive planning to limit risk and impact. Remotely-sensed data on terrestrial characteristics and variables such as temperature, rainfall, humidity, along with other environmental factors, govern the distribution of infectious agents as well as the intermediate hosts and vectors they might utilize. Therefore, the application of this new technology has been growing rapidly and nowhere is this more evident than with regard to parasitic diseases [11].

The application of geospatial activities summarized here emanates originally from a team residency in April 2000 at the Bellagio Study and Conference Center in Italy, sponsored by the Rockefeller Foundation. It was dedicated to the development of computer-based models for improving control programmes for schistosomiasis and other snail-borne diseases of medical and veterinary importance, and resulted in the establishment of the 'Global Network for Geospatial Health' whose scope has since expanded to include also other widespread infectious diseases [1]. The current objectives of the network are to:

• facilitate the development of GIS-based early warning systems concerning infectious diseases;

• collect data, produce risk maps, develop ecological niche models, and distribute GIS health applications of general interest via its website;

• offer training courses on health applications of geospatial tools through an 'International School of Geospatial Health'; and

• publish *Geospatial Health*, an open-access, peer-reviewed journal that focusses on health applications in the geospatial sciences, which accepts articles on all aspects of the application of GIS, RS and other spatial analytical tools in veterinary and public health.

Zoonotic diseases, in particular parasitic diseases with their dependence on intermediate hosts and/or vectors, are closely linked to environment, ecology and climate [12,13]. Therefore, the spatial distribution of these diseases can reveal important information of their transmission. The symposium emphasized spatial techniques and spatial analytic methods. GIS, global positioning systems (GPS) and RS belong to the general toolbox of any studies in this field, while spatial statistics (spatial point pattern analysis and clustering analysis) lends itself particularly well to the study of schistosomiasis and leishmaniasis as shown below.

## General approach

In the developing world, parasitic infections by multiple species are the norm rather than the exception [14]. In view of the growing political and financial commitment to control these parasitic diseases, there is a need to develop spatially-explicit databases so that control efforts can be targeted in an efficient and cost-effective manner [15,16]. Therefore, the symposium had, as the main focus, the establishment of spatial databases for risk profiling of multiple species parasitic diseases. The introductory discussion illustrates how demographic, environmental and socio-economic data can be readily obtained from existing village registries, simple cross-sectional questionnaire surveys and remotely-sensed environmental data obtained from satellite sensors [17]. The information sought is very often available in the form of ground maps, aerial photos, satellite images, whereas geographical coordinates are readily obtained by a GPS tool. When official censuses cannot be obtained, the population data, including demography and socio-economy, in the confined area can be supported by questionnaires. Infection and/or disease, including clinical, parasitological, serological and morbidity data, can then be presented in an overlaying raster, incorporated into a GIS platform and linked to socio-economic and/or environmental data derived from cross-sectional surveys and remote sensing data. Accurate information on multiple species parasitic infections requires adequate sampling and the use of a combination of different diagnostic methods. With such spatially-explicit databases at hand, researchers can perform risk factor analyses and produce risk maps of both single and multiple diseases [17-19].

## Technological aspects and methodology

### Geo-referencing and visualization

GIS has become a universally applied platform for visualization and analysis of spatial data, while GPS and RS have become important tools to obtain geographic information in epidemiology [16,20,21]. Accuracy of positioning is an important GPS characteristic and was indeed widely used in the papers presented at the symposium, particularly in small-scale field work where particularly accurate positioning is needed. Qing Yu introduced the symposium participants to a wireless GPS capable of communicating positions in real-time, rapidly recording information and maintaining a close contact with the monitoring center [22].

RS is an important data resource for presentation of vegetation, land cover and land use. Typically, mosquito and snail habitats are closely related to these factors which therefore make necessary ingredients in studies exploring the density of vectors and intermediate hosts of parasites [23,24]. Xinjian Xu and colleagues discussed the use of RS image manipulation to study the potential expansion of the snail-infested areas with the 1998 unusual flooding of the Yangtze River [25]. They found areas suitable for habitation by *Oncomelania hupensis*, which occupied 23.3%, 40.1%, 43.1% and 5.7% in total of the flooded areas in Jiang Han Plain, Dongting Lake, Poyang Lake and the lower reaches of the Yangtze River in Anhui province, respectively. This type of area could rapidly translate into high-risk areas for schistosomiasis.

Lingling Ma retrieved eco-environmental factors concerning *Oncomelania *snail distribution from QuickBird satellite images of Pengxinzhou, Dangtu county, Anhui province. She constructed a spatial GIS database of snail distribution based on an ArcGIS platform from 153 ground observations, which includes snail density, the normalized difference vegetation index (NDVI), the modified soil adjusted vegetation index (MSAVI), the leaf area index retrieved by NDVI (LAI_NDVI_), the leaf area index retrieved by MSAVI (LAI_MSAVI_), the vegetation cover ratio retrieved by NDVI (F_NDVI_) and the vegetation cover ratio retrieved by MSAVI (F_MSAVI_). Based on these measurements, a regression formula could be articulated modeling the distribution of *O. hupensis *based on the collected data as follows:

*Y *= -3.92 + 1.22 × *LAI*_*MSAVI *_+ 16.08 × *F*_*MSAVI*_

where *Y *is the snail density, *LAI*_*MSAVI *_the leaf area index and *F*_*MSAVI *_the vegetation cover ratio retrieved by MSAVI [26]. Analysis of remotely-sensed images can also help to classify the habitat type. For example, Zhaojun Li and colleagues found five different types of environment in the high-risk marshlands around the Poyang Lake, namely (i) water, (ii) sand, (iii) swamp, (iv) flat beach, and (v) steep beach [27]. The latter three types can be remotely-sensed and identified by the TM satellite and can thus be utilized to get an idea of the vegetation of the marshlands. RS analysis can, in principle, spatially confirm the distribution pattern of *Oncomelania *snails, i.e., their habitats are mainly distributed in the vegetation areas of lower lake-beach locations. In this way, high-risk endemic areas can be clearly, precisely, and quickly located by superimposing vegetation data onto a general map of an area.

In China, geospatial techniques have been applied to research on schistosomiasis in the plain and lake regions of China, but few of this has been previously attempted in the mountainous regions of Sichuan and Yunnan provinces [28]. It was therefore of great interest to note the work by Yi Dong and his group in Dali, Yunnan province. They showed, for the first time, that the altitude is an important factor affecting the snail density in this type of endemic area, using the following formula:

*Y *= log^-1 ^(1.12 + 2.14 × *X *- 0.001 × *X*_*1*_)

where *Y *is the snail density, *X *the NDVI and *X*_*1 *_the altitude above sea level in meters [28].

Spatio-temporal clustering analysis is usually used to explore the epidemic tendency of infectious diseases [29]. Zhijie Zhang and Qing Fu provided examples of this in their studies on schistosomiasis and visceral leishmaniasis, respectively. In the schistosomiasis study, two clusters with statistical significance in the Guichi district, Anhui province were found [30]. The leishmaniasis study area was located in the oasis area in Kashgar (or Kashi) region in China, which is a focus for control, prevention and surveillance of visceral leishmaniasis. In this study, Qing Fu and colleagues identified three spatial cluster zones [31].

Interpolation, kriging especially, is useful for the production of the predicted value at unknown locations based on the known values of surrounding points. In epidemiological surveys, it is helpful to grasp the overall situation by acquiring information about non-sampled locations. Shan Lv applied this approach to study the prevalence of *Angiostrongylus cantonensis *in China [32]. This parasite, causing meningoencephalitis, was identified in the intermediate host snails collected from the fields and it was shown that two invasive snail species (i.e., *Pomacea canaliculata *and *Achatina fulica*) had become important vectors/hosts.

Although, GIS is useful for the analysis of spatial patterns and spatial associations, as well as a repository for storage, management and visualization of spatially-explicit data, most software packages lack the simulative and predictive capabilities necessary for complex dynamic processes such as infectious disease transmission. Haitang Hu and colleagues discussed this aspect introducing a process-oriented spatio-temporal model for schistosomiasis transmission simulation [33]. To represent not only objects and events, but also processes and their relationship, they proposed integration of four base classes besides the object classes in traditional GIS, namely (i) a parameter class, (ii) a model class, (iii) a process class, and (iv) a process-set class. To effectively represent the output of spatio-temporal analysis and process simulation, they developed a series of visualization methods including maps, graphs, tables, animations (map or graph-based) as well as internal links and interactions. The model supports visualization of the spatio-temporal information for one or more geographically dynamic processes which can be simulated through animation. Users can browse any status instantaneously by setting the time and they can also explore continuous change of any of the process of interest in the form of animation.

### Data management and web-based databases

The integration and accessibility of data are common problems for all aspects of modern research, especially for a deeper understanding of parasitic and pathogenic diseases, where a variety of details for patients, hosts, parasites, vectors/intermediate hosts and environmental conditions are required for epidemiological studies.

The web-based public health and disease control GIS system in the Mekong region is a good example to exhibit how to gather, collate, visualize and analyze data. Zhang discussed the design and implementation of the 'Mekong Disease Control Web GIS', based on the 'China Next Generation Internet' (CNGI) [34]. In order to test web application in this new CNGI environment, GIS for public health and disease control has been built into the project which includes six countries related to the Mekong River. The system is used for the sharing of public health and disease control information in this region and consists of CNGI hardware containing Internet protocol version 6 (IPV6) gateways and switches and a WebGIS software, which provides service and client support [35]. In this system, the database includes five parts, namely (i) basic geographic data, (ii) sanitation data, (iii) statistics, (iv) epidemic disease source data, and (v) outbreak epidemic data. The three main functions include public health and disease information, epidemic reporting and an emergency command structure. The outbreak epidemic disease data can be entered into the web system and deliver disease information in real time.

Another web-based system presented during the symposium is the Fireflower , which is equipped with multi-disciplinary electronic data collection and management service (including a language translation module) for use anywhere in the world at any time. Although Fireflower can be used by individuals, it is particularly useful for interdisciplinary research teams, where members may work from a wide variety of different locations. Data can be collected at any time from laboratories, clinics, hospitals or the field, and immediately made available to other members of the team, wherever they are. Fireflower is currently being used by the EU-funded CONTRAST programme, a multidisciplinary alliance to optimize schistosomiasis control and transmission surveillance in sub-Saharan Africa . Within this programme, detailed schistosomiasis data are collected from different countries across sub-Saharan Africa in a collaborative venture and historical data are retrieved from systematically reviewing the extent literature [36]. The system enables data from many different aspects to be integrated, correlated and analyzed, and provides simple mechanisms for searching, sharing and dissemination of information to authenticated clients. The system works in conjunction with GIS systems and a particular useful aspect is that users can toggle between different languages and fully understand each other without personal multi-language knowledge.

### Modelling and Bayesian geostatistics

The way Bayesian statistics facilitates geospatial and epidemiological analysis has made it increasingly popular [17,37-40]. In order to understand the spatio-temporal heterogeneity of schistosomiasis, Xiao-Nong Zhou and Kun Yang applied this type of statistics to schistosomiasis transmission dynamics to identify effective intervention strategies in different settings [41]. They investigated the epidemiology at various spatial scales by developing spatio-temporal models to analyze county-level data from serological surveys carried out in China from 2002–2005 by taking into account NDVI and land surface temperature (LST) data from the MODIS satellite. They also used an index indicating the prevailing economic development for the area studied. This work showed that the impact of environmental factors on seroprevalence of *Schistosoma japonicum *infection varied in accordance with the environment and economic status of study villages. In the lake regions, there was a strong spatial correlation with temporal variability of seroprevalence and the spatial correlation coefficients ranged from 0.87 to 0.95 [42]. Places with relatively high prevalence rates were concentrated near the Poyang and Dongting lakes, while, in the mountainous endemic regions, the spatial correlation was weak. Furthermore, in a study in Dangtu county, it was found that a second-order ordinary kriging approach of spatial analysis facilitated the prediction of human prevalence of *S. japonicum *infection at the unit of the village. Four different strata, based on human prevalence rates and type of transmission, were established. Subsequently, different strategies to control schistosomiasis transmission, based on environmental idiosyncrasies, were put forward [43]. The use of landscape pattern analysis, coupled with Bayesian spatial modeling, was successfully applied to predict the distribution of *O. hupensis *at the local scale [41].

A study, offering insight into a potential impact of climate change on mosquito abundance due to higher tide frequencies and intensities with the expected rise of the sea level, showed by Guo-Jing Yang and colleagues that efficient and effective mosquito control requires a better understanding of vector population dynamics and how these are modified by endogenous and exogenous factors. A dataset describing the relative abundance of *Aedes vigilax *in the greater Darwin region, northern Australia, over an 11-year period was examined in a suite of Gompertz-logistic (GL) models [44] with, and without, hypothesized environmental correlates (e.g., high-tide frequency, rainfall and relatively humidity). It was also examined whether environmental correlates could explain the variance in seasonal carrying capacity (*K*). Models were compared using Akaike's information criterion (AIC) [45], Bayesian information criterion (BIC) [46], and the so-called jack-knifed cross-validation (C-V) [47]. The GL model with a 2-month lag without environmental effects explained 31% of the variation in population growth rate [44]. This increased to >70% under various model combinations of high-tide frequency, rainfall and relative humidity. Temporal variation in *K *was explained by high-tide frequency and reduced *Ae. vigilax *carrying capacity over the study period. This investigation underscored the imperative of considering simultaneously both types of drivers (endogenous and exogenous) when predicting mosquito abundance and population growth patterns.

### Diagnostic techniques

Peter Steinmann and colleagues assessed different techniques for diagnosis of intestinal parasites in human faeces [48]. Up to three stool specimens from a random population sample in the Nongyang village in the south of Yunnan province were collected and subjected to four diagnostic techniques: the Kato-Katz method, an ether-concentration technique (only 1 sample/person), the Koga agar plate method and the Baermann technique. It was found that screening three rather than a single stool sample and employing different techniques increased the diagnostic sensitivity, most notably for hookworm and *Strongyloides stercoralis*. Furthermore, it was reported that *S. stercoralis *is endemic in the southern part of Yunnan province [48]. The authors concluded that the use of different diagnostic assays and the collection of multiple stool samples are necessary to estimate the 'true' extent of intestinal parasites and to investigate the often neglected issue of multiparasitism [48,49]. Researchers should therefore be encouraged to use a suite of diagnostic approaches to gain a more valid picture of local parasite fauna, which in turn can guide subsequent integrated control interventions.

### Molecular and genetic techniques

It would seem that the combination of genetic variation and geographic distribution should reveal biodiversity. To that end, Thanh Hoa Le mapped the spatial distribution of different food-borne and water-borne zoonotic parasites (i.e., *Fasciola *spp.,*Fasciolopsis buski*, *Opisthorchis viverrini*, *Clonorchis sinensis*, *Paragonimus heterotremus *and *Haplorchis *spp.) in Vietnam, based on sequences of mitochondrial DNA (*cox1*; *nad1*), nuclear internal transcribed spacer 2 (ITS-2) and alternatively, ribosomal 18S rDNA [50]. The molecular analysis revealed that the Vietnamese *Fasciola *population belonged to two genotypes: a pure *F. gigantica *and a hybrid/introgressed form of *F. gigantica *and *F. hepatica*, both distributed throughout Vietnam and found in all hosts [50]. Molecular analysis confirmed that opisthorchiasis in Vietnam comprises of *O. viverrini *(in the southern provinces) and *C. sinensis *(in the northern provinces). While *C. sinensis *showed little variation, *O. viverrini *indicated a broad divergence based on comparative analysis of isolates from Vietnam, Laos and Thailand. *Faciolopsis *spp. were molecularly identified as *F. buski *and, unlike *Fasciola *spp., genetically unique in isolates of human and pig origin in Vietnam but varied slightly in comparison to Indian samples. The *Taenia *population comprises *T. saginata *in cattle, *T. solium *in pigs and humans, and *T. asiatica *in human. In Vietnam, *T. solium *belongs to the Asian subgroup. *P. heterotremus*, collected from different life-stages and hosts in Vietnam, was found to be molecularly unique and seen only in hosts from the mountainous provinces of North Vietnam. The small intestinal flukes were identified as a mixed population of *H. pumulio *and *H. taichui*, with a possible intermediate genotype based on the variability of mini-satellites in the ITS-2 marker. The *Haplorchis *complex is distributed in the 15 provinces so far investigated in Vietnam. In addition, Thanh Hoa Le and colleagues have now completed the mtDNA sequencing for *C. sinensis*, *O. viverrini*, *F. gigantica*, *F. buski*, which will undoubtedly facilitate the molecular investigation of these food-borne trematodes [51].

Smarn Tesana analyzed the genetic variation of *Bithynia siamensis goniomphalos*, the first intermediate host of *O. viverrini*, from 19 provinces in northeast Thailand using RAPD-PCR [52]. Three localities in each province and 10 snail samples from each locality were collected for the study, 570 samples in total. The intra-population variability was studied in 10 ecological sites (10 snail samples from each site), looking for genetic homogeneity. The results were analyzed by using phylogeny inference package (PHYLIP) and a phylogenetic tree constructed with the snails in the same family of Bithyniidae (*B. siamensis siamensis*, *B. funiculata *and *Wattebledia crosseana*) as out-groups. The genetic variation of inter-population could be divided into four groups belonging to the basin of the four main rivers of the northeast, namely (i) the Mun River, (ii) the Chi River, (iii) the Songkhram River, and (iv) the Leoi River, while the genetics seemed to be relatively conserved in species from each of the separate water systems [52].

## Applications

### Food-borne diseases

Food-borne trematodiases, caused by *C. sinensis*, *O. viverrini*, and other liver, lung and intestinal flukes are truly neglected tropical diseases. They constitute a public health problem of enormous proportions: an estimated 750 million people are at risk for these infections with more than 40 million individuals currently infected [53,54]. Many factors conspire to explain their high prevalence, but lack of insight and poor recognition of these trematode infections, in the face of lacking political will and financial means to control them, may explain their neglected status [55]. In addition, the generally poor sanitation, poverty, malnutrition, exponential growth of inland fish production (aquaculture) and lack of food inspections in the endemic areas no doubt contribute substantially to the situation which, in fact, could be controlled with the tools available today.

At the symposium, survey data covering the period 1990–2006 were reported to have found 14% of examined people to be infected with *C. sinensis *in Guangdong province, China [56]. The distribution of these cases had a strong link to the water network of the Pearl River, which leads to an estimate of 5 million people possibly being infected in the province as a whole. Interestingly, although Anhui province in central China, which is also characterized by a prominent water network, had an infection rate below 1% (based on the 2002–2003 survey [57]). It is conceivable that the higher socio-economic development in Anhui province is an underlying factor explaining the lower *C. sinensis *infection prevalence compared to Guangdong province, a fact supported by the finding that the highest infection rates were found in remote rural areas such as, for example, the northern plain region along the Huaihe River.

*B. siamensis goniomphalos *is the first intermediate host snail of *O. viverrini *in Northeast Thailand. The snail's genetic variation has been analyzed and shown to be divided into four groups belonging to the basin of the four main rivers of this part of the country [52]. A reported high prevalence of '*Opisthorchis*-like' eggs in human faecal samples was reported from a study area in Laos [58]. In a survey covering two villages and some hospitals, 86% of general villagers and 90% of hospitalized patients, respectively, were found to be infected with *O. viverrini*. The distribution of the disease and the parasite in Southeast Asia clearly indicates that both are endemic throughout the region but there is a strikingly high prevalence of cholangiocarcinoma in northeast Thailand and Laos where this liver fluke infection is particularly common [59]. Indeed, there is no stronger link between a human malignancy and a parasitic infection than the link between cholangiocarcinoma and *O. viverrini *infection [60].

Cysticercosis, caused by *T. solium*, and cystic echinococcosis (hydatidosis) are often neglected in China in spite of their effect on public health and agriculture [61]. Echinococcosis is mainly endemic in pasture areas in the western part of the country and a recent survey showed that the prevalence of the disease is, on average, 1%, while serological antibody screening indicates that 12% have had contact with the parasite in the relatively recent past [62]. Tibet, Qinghai, Sichuan, Gansu, Ningxia, Xinjiang and Inner Mongolia are all highly endemic areas, while provinces such as Jilin, Heilongjiang, Shanxi, Shaanxi, Yunnan, Hebei and Henan have much less infection and most other areas have none at all. In recent years, the disease has spread to previously non-endemic areas and the number of imported cases has increased. In Nepal, the disease is prevalent in Banepa, Panauti and Dhulikhel [63]. Although the number of humans in the areas investigated is low, there are indications that 9% of the population is infected [64]. Several factors facilitate transmission such as uncontrolled slaughtering under unhygienic conditions, lack of meat inspection programmes, and unawareness of how the disease is transmitted.

*Taenia *infections are related to certain ethnic groups and their pork consumption habits, literacy rate, hygiene behaviour and lack of adequate sanitation. Pork consumption is rapidly increasing in Nepal and this influences the incidence of this infection. Among studied ethnic groups, Magars, Sarkies, Darai and Bote, the prevalence was found to be 50%, 28%, 10% and 30%, respectively [64]. A recent report claims that 10.5% of 200 slaughtered pigs were positive by lingual examination, while post-mortem examination, presumably more accurate, gave a positive rate of 20.5% [64].

Taeniasis and cysticercosis are highly prevalent in China's poor regions, e.g., the infection rate of *T. solium *has been found to be as high as 67% in Lanping county, Yunnan province [65]. A survey conducted in the Yi minority township in Sichuan province in 2006 found 9% of the population reporting epilepsy or occasional seizures, while subcutaneous nodules were observed in 1.5% of all people examined [66]. Although less than 1.5% actually excrete parasite eggs according to stool examinations, a PCR test performed on 151 of the 191 faecal samples tested, found traces of *T. solium asiatica *in 62 individuals (41%) and in one person (0.7%) traces of *T. solium asiatica *[66]. Several risk factors appeared to be important in this community, including common consumption of raw or undercooked pork and absence of pig fencing. Additionally, latrine-facilities commonly serve many families. Thus, poor hygiene conditions, low socio-economic status and little knowledge about this disease facilitate the transmission.

Angiostrongyliasis is an emerging parasitic disease in China and public awareness increased when a large outbreak occurred in Beijing in 2006 with 160 individuals involved [67]. The first national survey covering the endemic areas was conducted in 2006 and revealed that natural transmission of angiostrongyliasis are particularly common in the Fujian, Guangdong, Guangxi, Zhejiang, Hainan, Hunan, and Jiangxi provinces. Importantly, *A. cantonensis *was identified in two invasive snails species (i.e., *P. canaliculata *and *A. fulica*) [32,67]. It has been proposed, based on infection rates of *P. canalicualta*, that the endemic areas should be classified according to the degree of endemicity and preventive measures implemented according to the need (Table 1).

**Table 1:** Preventive measures proposed for different types of endemicity regions for angiostrongyliasis

**Degree of endemicity **	**Preventive measures proposed **
Hyper-endemic area	Public health education and training of medical practitioners to strengthen market management and food-safety. Introduction of a case-reporting system.

Meso-endemic area	Strengthening of general surveillance with monitoring of markets, breeding farms and restaurants.

Hypo-endemic or potentially endemic areas	Occasional sampling and surveys in suspected places

### Aquaculture

**Figure 1:**
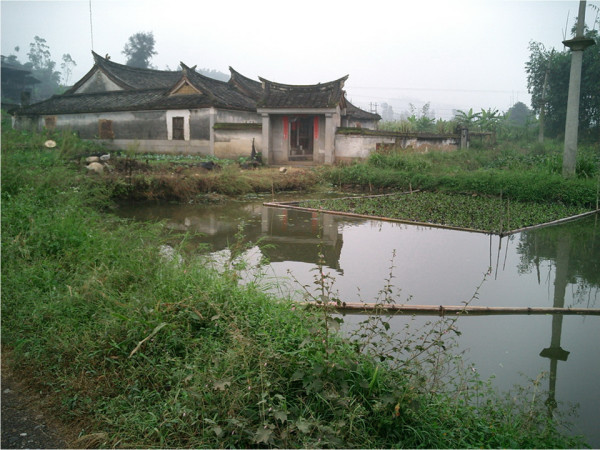
**Photograph showing a typical fish pond used for acquaculture in the region of Southeast Asia**.

**Figure 2:**
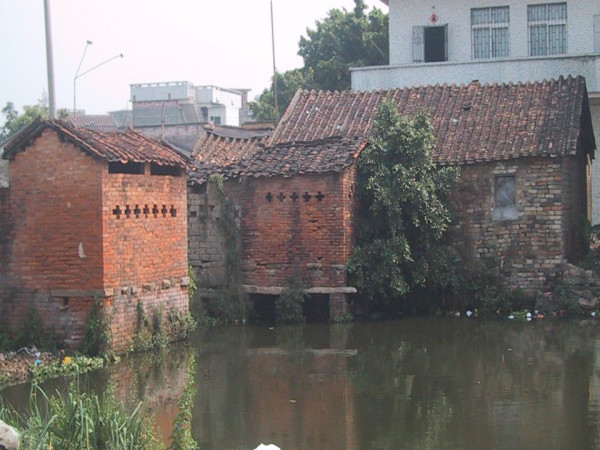
**Photograph showing the natural environment where transmission of *Clonorchis sinensis *occurs**. (i.e., traditional latrine located on the top of a fish pond used for acquaculture)

The increasing prominence of aquaculture in Southeast Asia, with several-fold increases in freshwater fish production in the last few decades [54], have emphasized the role of aquaculture in the transmission of fish-borne zoonoses. In Vietnam, a large project, the "Fish-Borne Zoonotic Parasites in Vietnam Project (FIBOZOPA)"  involves Vietnamese and Danish institutions and has been launched to foster research and build capacity to assess the status and risks for fish-borne zoonoses in Vietnamese aquaculture [68]. The project started in 2003 and has documented widespread presence of fish-borne zoonoses in aquaculture, including seven new fish-borne parasites in Vietnam [68]. New evidence has been generated for the important role of domestic animal reservoirs in sustaining fish-borne zoonoses in the absence of human infection. Work is in progress on identifying snail intermediate hosts and assessing the ecological factors regulating their populations as well as the development of molecular-based detection tools. Future research needs to establish sustainable fish-borne zoonoses control measures in Vietnam and nearby countries such as Cambodia, Laos and Myanmar. These would include mapping out the national and regional distributions of fish-borne zoonoses determining underlying risk factors for transmission (Figures 1 and 2).

### Soil-transmitted helminthiases

#### Hookworm infection

Hookworm infection is a major cause of anaemia and malnutrition of humans living in the developing world, especially in the tropical regions [69,70]. The prevalence varies with the geographical location as well as with the prevailing economic and social conditions. Recent studies in Yunnan province, China [48,71] showed a prevalence as low as 0.3% in Dali, while the level was 89% in Xishuangbanna, a community which is not far away from there. In one survey in Hainan island, the southernmost Chinese province, the overall infection rate of hookworm was found to be 33% [72]. A trend of increasing incidence with age was seen and the infection rate in farmers was higher than that of other occupations. In addition, the highest rate was found in the mountainous area on the island. Species identification showed that 94% of the infected persons harboured *Necator americanus *with less than 1% infected with *Ancylostoma duodenale *only, while mixed infections accounted for 5% of all cases. In 2003, the overall prevalence in Yunnan province was shown to have decreased from 22% in the early 1960s to less than 6% and it is expected to be even lower today [73].

#### Ascariasis

Ascariasis, caused by *Ascaris lumbricoides*, is the most common helminthic disease globally, with an estimated worldwide prevalence of 25% (0.8–1.4 billion people infected) [74,75]. Usually asymptomatic infections are most prevalent in children in the tropical and developing countries, where they are perpetuated by direct contamination of the soil by human faeces or the use of untreated faeces as fertilizer ('night soil'). Symptomatic disease may be manifested by growth retardation, pneumonitis, intestinal obstruction, or hepatobiliary and pancreatic injury. In developing countries, ascariasis may exist as a zoonotic infection associated with exposure to pigs or pig manure. In China, the prevalence of *A. lumbricoides *infections have come down from 65.6% in the early 1960s to 16.9% in 2003 in Yunnan province [73], but it is still very high in some regions and remains therefore an important public health problem. A recent survey, conducted in the village of Nongyang in the southern part of Yunnan province, found an *A. lumbricoides *prevalence of 92.6% [48].

#### Trichuriasis

Trichuriasis, caused by *Trichuris trichiura*, is the third most common roundworm infection of humans [76]. Like ascariasis, the disease occurs globally, with infections more frequent in areas with tropical weather and poor sanitation practices, and often among children [74,75]. It is estimated that 800 million individuals are infected worldwide [74,75]. However, in Yunnan province, the average prevalence of *T. trichiura *was reduced from 12.9% in the early 1960s to 2.2% in 2003 [73]. Steinmann and colleagues recently reported the prevalence of *T. trichiura *to be 1.7% in Dali and 88.8% in Xishuangbanna prefecture in Yunnan province [48,71].

#### Other soil-transmitted diseases

In the above-mentioned survey conducted in the village of Nongyang in Xishuangbanna prefecture, sufficient amounts of stool were obtained from 180 participants for the appraisal of *S. stercoralis *using two diagnostic tests and the prevalence was found to be 11.7% [48]. *Enterobius vermicularis *was found in 7.4% of the participants in the same community-based survey [48].

### Water-borne parasitic diseases

#### Schistosomiasis

Although many water-borne, intestinal protozoa capable of causing important infections exist in Southeast Asia (as well as in most parts of the tropical world), e.g., *Blastocystis hominis*, *Endolimax nana*, *Giardia intestinalis*, *Entamoeba coli*, *E. hartmanni*, *E. histolytica/E. dispar *and *Iodamoeba bütschlii*, their role is greatly overshadowed by schistososomiasis, which is caused by *S. japonicum *or *S. mekongi *in this part of the world [77]. In Southeast Asia, schistosomiasis is a main threat to health in China and the Philippines and exists focally also in Indonesia, Cambodia and Laos.

Few countries have achieved as great a success in schistosomiasis control as has China [78]. The control programme has been sustained for over 50 years and the human prevalence rate of *S. japonicum *has gradually been lowered year by year resulting in a more than 90% decrease over this period of time [79]. However, even if the prevalence has been greatly reduced in most of the country, interruption of transmission remains a formidable challenge [80,81]. In order to provide information on the significance of bovines in the transmission of human schistosomiasis, the spatial and temporal contamination of the environment by bovine faeces has been studied [82]. Xiao-Hua Wu and collaborators performed correlation and regression analyses using data from three national sampling surveys on schistosomiasis, carried out in 1989, 1995 and 2004, respectively, established a GIS and performed spatial analyses to identify the high-risk areas of the disease [83]. They found that while the human prevalence and force of transmission in highly endemic areas (e.g., in marshlands along the Yangtze River and in the southwestern mountainous region [84]) has been reduced since 1989, the relative importance of bovine schistosomiasis has increased. This is reflected by a declining Spearman correlation coefficient between the infection prevalence in humans and in bovines over time (0.81 in 1989, 0.75 in 1995 and 0.38 in 2004). In parallel, the slope of the linear regression decreased from 0.39 in 1989 to 0.21 in 2004 [83]. The data clearly suggest that future schistosomiasis control efforts in China should more vigorously address the important role of bovines in the transmission of human schistosomiasis [85], and focus on the reduction of the environmental contamination of *S. japonicum *eggs by bovines [86].

**Figure 3:**
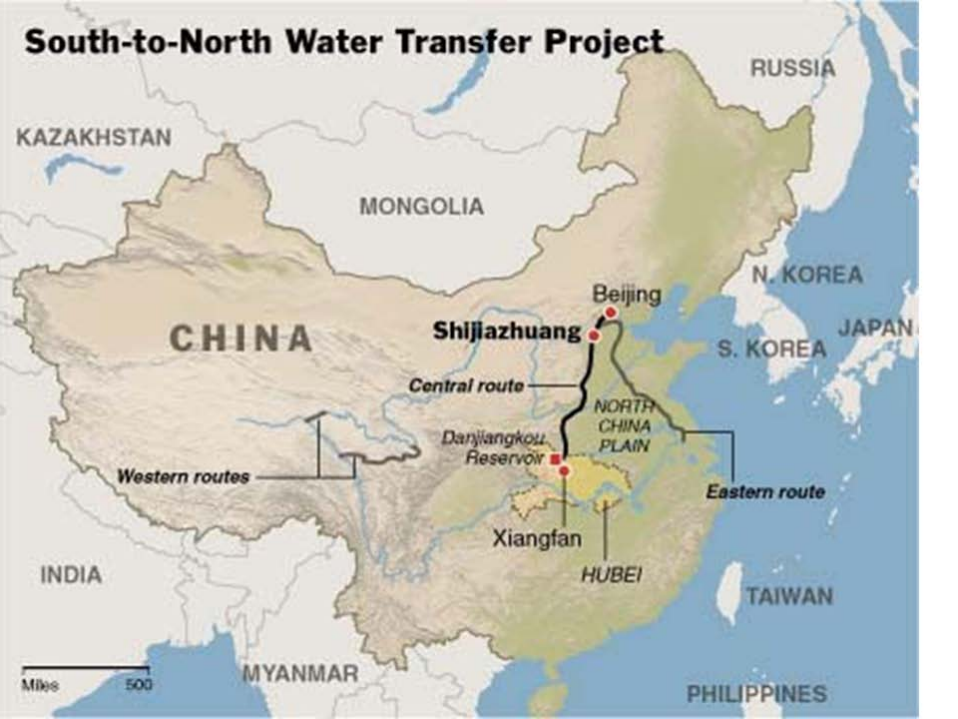
**Map showing the routes of the South-to-North Water Transfer Project in China**.

Less well-known, but still the largest water deviation in the world, is the 'South-to-North Water Transfer Project' (SNWTP), aimed at improving the current water shortage in the northern part of China [87,88]. It adds to the risk posed by bovine schistosomiasis by opening the gates for the dispersal of infected O.*hupensis *snails along the diversion routes (especially the eastern routes) to northern China. Yixin Huang and colleagues evaluated the possibility of the endemic areas extending from the southern to the northern parts of China following the implementation of the SNWTP [89]. The researchers concluded that the water flow and constructions are secure vis-à-vis the spread of *Oncomelania *snails from the south to north in the water diversion of the eastern route project. However, the potential for transmission of *Oncomelania *snails may exist south of latitude 33°15 N in Jiangsu province, such as in the Lixia River basin, in the Jinbao River and in the Hongze Lake [88] (Figure 3).

Major ecological transformations occurred after the Three Gorges Dam construction across the Yangtze River [90-92]. Due to the high water velocity in the river before the construction, there was no natural *Oncomelania *habitat and, historically, there has never been any schistosomiasis transmission in this area. This situation has now changed and it was reported at the symposium that *Tricula sinica*, a snail which shares the ecological habitats with *O. hupensis*, has been discovered in the area [93]. The increase of fishing, brought about by the development of the economy in the Three Gorges Reservoir areas, has attracted mobile people who live in their boats on the reservoir. The investigation by Cheng-Guo Wu and colleagues [94] show that 66.8% of mobile population came from endemic areas and 52% of local residents returned from such areas had a history of contact with snail-infested-water. The sero-positive rates of the people, who came or returned from endemic areas, were 2.1% and 1.0%, respectively. An estimated 3.3% of these people came from endemic areas, and 1.3% of the individuals returning from them had a history of schistosomiasis. Additionally, more than 120,000 livestock were imported from endemic areas. Since there is currently no schistosomiasis transmission in the Three Gorges region, disease-specific health education is not regularly provided and less than 5% of the local residents know anything about schistosomiasis and its control [94]. All these factors conspire to increase the risk of introduction of schistosomiasis here and hence there is a pressing need for monitoring and surveillance.

The risk for transmission of schistosomiasis is determined not only by individual behaviour and water contact patterns, but also by village-level factors [95,96]. In the autumn of 2000, surveys were carried out in 20 villages in the Anning River valley in south-western Sichuan, an area with historically high rates of *S. japonicum *infection. It was found that 29% of more than 3,000 residents tested positive for schistosomiasis. A random-effects model allowing intercept of each village was used to estimate the effect of water contact on infection based on 1,011 people with infection and water-contact data. Each 100 min/m^2 ^increase in water contact turned out to increase the odds for infection by 1.1 (95% confidence interal: 1.0–1.2). A similar random-effects model was also used to examine how occupation, gender and age predicted an infection. Farming was found to be the strongest predictor with female farmers being at the greatest risk. In both analyses, the random-effects models produced a significantly better fit of the data than models lacking the random-slope factor, underscoring the importance of accounting for the variance in infection risks between villages when assessing individual-level predictors for infection [95,96].

The ubiquity of acute cases is important for judging whether a region has reached the criteria of schistosomiasis control, and is one of the sensitive indices to evaluate the epidemic situation [84]. The number of acute cases has become very small translating into reduced importance in the overall control efforts. However, the spatial statistics applied in recent years has made it possible to study the epidemic characteristics of these cases, which may provide a better support for the decision-making at the government level [97]. Zhijie Zhang *et al*. [30] analyzed the surveillance data of acute schistosomiasis using a spatial point pattern approach in the Guichi district, Anhui province and found that the geographic center of the distribution of acute cases shows a tendency to move southward. In this study, two clusters with statistical significance were identified. The most likely cluster was located in the intersection of the Yangtze and Qiupu rivers at latitude 30.65 N and longitude 117.44 E with a radius of 2.69 km. The relative risk was 12.78 (log-likelihood ratio = 32.80, P < 0.001). The coordinates of the second cluster, situated in the southeast of Guichi district, was latitude 30.34 N and longitude 117.61 E with a radius of 4.57 km and a relative risk of 8.76 (log-likelihood ratio = 11.15, P = 0.002). Identification of such clusters is important to prioritize public health planning and resource allocation for schistosomiasis prevention and control.

Despite the availability of effective drug treatment, schistosomiasis remains prevalent in the Philippines and re-infection is still a major issue [98]. Luz Acosta and her group assessed the impact of spatial clustering at the household level in predicting intensity of re-infection with *S. japonicum *[99]. They assigned clusters using the equal population area (EPA) method and compared their intensities of re-infection while adjusting for age, gender, baseline infection intensity, observed water contact, and socioeconomic status. The results show (i) that proximity to irrigation canals and man-made reservoirs predicts re-infection intensity, and (ii) that spatial clustering at the household level predicts intensity of re-infection after adjusting for age, gender, baseline infection intensity, and observed water contact. Spatial clustering of households is an indicator of exposure to sources of infection.

#### The intermediate host snail

*O. hupensis *is the sole intermediate host of *S. japonicum *in China. The availability of suitable habitats determines its distribution [8]. Propagation and multiplication of the snail are closely correlated to environmental factors such as temperature, humidity, vegetation and soil which make GIS and RS well suited to determine the location of potential snail habitats [97,100,101]. Snail control was once considered as one of the most important measure in schistosomiasis control in China [41,102], while chemotherapy has now become more prominent. The distribution of the snail intermediate host is far more extensive than that of the disease-endemic areas, supporting the need for snail control as an important part of the work for elimination of schistosomiasis in China. In fact, 14.6% (735) townships in China are non-endemic with regard to schistosomiasis but this status has the potential of rapid change as they still harbour the snails [82].

An experiment investigating the susceptibility of *Oncomelania *snails from a non-endemic area in Ningguo city, Anhui province to *S. japonicum *miracidia was reported. Surprisingly, it was found that these miracidia failed to infect the snails from the non-endemic area, while the snails from endemic areas could be infected [82]. This is an interesting result and, since no large study on the susceptibility of *Oncomelania *snails has been conducted so far, more experiments are urgently needed. If *S. japonicum *miracidia will be found to be non-infective to certain snails, or if the resulting infection rate is very low, the schistosomiasis control strategies will have to be modified.

Shanghai used to be an endemic area (before 1985) but is now free from schistosomiasis transmission. The result of 21 years of surveillance (1986–2006) showed that this status can be maintained in spite of immigrating infected individuals, who can be found all over the city in conjunction with *O. hupensis *snails. Three types of snail geo-distribution have been described in nine districts (Table 2). Thirty-six snail-infested localities were found in three districts since 2006, 58.3% of which were smaller than 100 m^2^, 33.3% had a size between 100 m^2 ^and 500 m^2^, and 8.3% were larger than 500 m^2^. Preventive measures such as sanitary disposal of human excreta, treatment of infected persons, treatment or elimination of reservoir hosts, thus remain important. In Shanghai, the snail population is kept low by niclosamide treatment [103].

**Table 2:** The classification of 9 previously endemic districts for schistosomiasis in Shanghai, China

Type	Current epidemic status	District name
I	No intermediate host snails found, although the ecological conditions are suitable for snail survival	Minhang, Jiading, Baoshan

II	Intermediate host snails found occasionally	Pudong New District, Nanhui, Qingpu

III	Intermediate host snails found from time to time	Jinshan, Songjiang, Fenxian

### Arthropod-borne infections

#### Leishmaniasis

The first confirmed case of visceral leishmaniasis in China was reported in 1907 [104]. Since then extensive areas north of the Yangtze River (including 16 provinces) have been recognized as endemic for visceral leishmaniasis [105]. The disease was especially rampant in the east, including the provinces of Shandong, Jiangsu, Anhui, Henan and Hebei. In the northwest, the disease was mainly prevalent in Shaanxi, Gansu and Xinjiang. Sporadic cases occurred in Beijing, Liaoning, Hubei, Shanxi, Sichuan, Ningxia, Qinghai and Inner Mongolia. About 530,000 cases were reported in 1951 [106]. According to surveys made in 1951, the average incidence in different provinces was 10–50 per 10,000 people. Since the launch of the nationwide control campaign against kala-azar in 1950–1958 [107], the disease was under control in most of the endemic areas. However, transmission was not interrupted in the mountainous and desert region where sporadic cases continue to be reported. Currently, visceral leishmaniasis is still prevalent or sporadically distributed in six western provinces/autonomous regions, including Xinjiang, Gansu, Sichuan, Shaanxi, Shanxi and Inner Mongolia [108].

Kashgar in Xinjiang autonomous region is the most prominent endemic region with respect to visceral leishmaniasis and the study by time-space clustering of the epidemic situation of the diseases there has already been mentioned before. With respect to visceral leishmaniasis and the three spatio-temporal clusters (A, B and C) identified by Qing Fu are of great interest. Zone A included Boshikelam, the Haohan township of Kashi city and Awati town in Shufu county. Its centre was at latitude 39.52 N and longitude 76.08 E with a radius of 6.58 km. The high-risk time frame, calculated by kriging based on the ArcGIS software, was from 1 January, 1999 to 31 December, 2003. The relative risk of visceral leishmaniasis incidence was 46 times than that of outside of the scope (P < 0.001). Zone B involved reclaimed farmland of Bachu county with its centre was at latitude 39.91 N and longitude 79.20 E with a radius of 4.93 km. The high-risk time frame was from 1 January, 2002 to 31 December, 2006 with a relative risk of visceral leishmaniasis incidence 9.6 times higher than outside the scope (P < 0.001). Zone C included Yinwusitang town in Shufu county and Yangdaman town in Shule county. Its centre was at latitude 39.40 N and longitude 76.23 E with a radius of 7.63 km. The high-risk time frame was from 1 January, 2000 to 31 December, 2004, and the relative risk of visceral leishmaniasis incidence was 5.2 times higher than outside the scope (P < 0.001).

Four epidemiologic and clinical types of leishmaniasis have been recognized in China [109]. First, the Indian type of classic kala-azar is anthroponotic (human to human), caused by *Leishmania donovani *and most of the patients are adults. This type is mainly distributed in the plains of eastern China where the vector is the domestic *Phlebotomus chinensis*, and in old oases in Kashi, Xinjiang, where the disease is transmitted by peridomestic *Ph. longiductus *[106]. Second, the Mediterranean type of infantile visceral leishmaniasis in the northern, central and western hilly regions, caused by *L. infantum *and transmitted by wild or semi-wild species *Ph. chinensis*. Dogs constitute the most common reservoir and infants and children under 10 years of age are predominately the patients. Third, the desert type of visceral leishmaniasis in the northwest region is mainly distributed in Xinjiang, Inner Mongolia and Gansu provinces/autonomous regions. This disease is caused by *L. infantum *and some unknown species of wild animals and transmitted by wild species *Ph. wui *and *Ph. alexandri*. Infants and children under three are commonly the victims. Fourth, cutaneous leishmaniasis in the western desert area is only found in Karamay, Xinjiang autonomous region. It is caused by *L. infantum*, transmitted by wild species *Ph. wui*. A 3-year survey from 1992–1994 indicated that the annual morbidity of cutaneous leishmaniasis in Karamay was 1.6% (36/2260), 1.0% (14/1416) and 1.6% (24/1510), respectively [109].

Additionally, two species of *Leishmania*, nonpathogenic to humans but which can infect the great gerbil, *Rhombomys opimus*, have been found in China: *L. gerbilli *and *L. turanica *[110]. The former is found in Xinjiang, Gansu and Inner Mongolia and the vectors are *Ph. mongolensis *and *Ph. alexandri*, while the latter is in Karamay of Xinjiang where the infection is transmitted by *Ph. mongolensis *and *Ph. andrejevi*. Inoculation of the parasite in humans induces a self-healing ulcer.

Visceral leishmaniasis is still endemic in some mountainous areas in Sichuan province. Infected dogs and patients with *L. donovani *are major infection sources. *Ph. chinensis *as known sole vector is a wild type inhabiting the mountains. Although the disease has been under control since the founding of the People's Republic of China in 1949, leishmaniasis has gradually reemerged since 1972. The current endemic area covers six counties in northwestern Sichuan, including Jiuzhaigou, Heishui, Maoxian, Wenchuan and Lixian county of Aba Tibetan/Qiang Nationality Autonomous prefecture, and Beichuan county of Mianyang municipality. During 2003–2006, a total of 124 cases were reported in the endemic areas, mainly detected in the Jiuzhaigou and Heishui counties accounting for 86.4% of the total [111]. Pre-school and school-aged children are generally the victims. Currently, the following strategies are carried out: treatment of patients, health education, dog registration and management, and sandfly control in combination with sanitary movements of cities or towns. It is also crucial to pay attention to immigration from non-leishmaniasis-endemic regions.

Two presentations of the *Leishmania *problem in Europe discussed the spatial distribution of leishmaniasis in Italy. The classical visceral leishmaniasis endemic zones are the Tyrrhenian littoral, the southern peninsular regions and the islands, where *P. perniciosus *is the main vector. Human visceral leishmaniasis occurred sporadically with fewer than 40 cases per year through the 1980s, but since the early 1990s, the incidence has steadily increased with cases reported from throughout the country and reaching over 200 cases per year in the new millennium [112]. Beside the risk of visceral leishmaniasis spreading among HIV-positive individuals, circumstantial evidence suggests that the disease is actually spreading into previous non-endemic territories [113]. Studies covering the last five years indicate that the infection in dogs has increased from 21% to 32% from 2002 to 2005 and recent research shows that the prevalence exceeds 40% around the Vesuvius mountain [112,114].

In order to review and monitor this evolving scenario, surveillance activities have been implemented in north Italy, by including (i) an analysis of all human visceral leishmaniasis cases recorded in the 1990–2005 period; (ii) a retrospective literature analysis of all canine leishmaniasis cases and sandflies records through 2002; (iii) prospective serological investigations in dogs from areas where autochthonous canine leishmaniasis cases were confirmed (2003–2005); and (iv) surveys of the phlebotomine vector performed during the 2003–2004 warm seasons in the above areas. The results retrospectively identified seven foci of canine leishmaniasis since 1990, whereas the prospective investigation disclosed 47 autochthonous cases and 106 autochthonous seropositives among 5,442 dogs (2.1%) from 16 foci of six regions [114]. Four vector species were identified among 1,696 *Phlebotomus *(*Larroussius*) specimens collected which indicates that *P. perniciosus *and *P. neglectus *have increased in density and expanded their geographic range in the study area [112]. These findings demonstrate that northern continental Italy is now focally endemic for visceral leishmaniasis and that a moderate risk for human disease does exist there. However, the intensity of transmission seems to be lower than in traditional settings of Mediterranean visceral leishmaniasis.

#### Malaria

Malaria is common in Southeast Asia and the problem is particularly pronounced in the border regions between countries with limited health resource, where poor sanitary conditions prevail and multi-drug resistant parasites are found [115-117]. The Yunnan province, located adjacent to Vietnam, Laos and the Mandalay area, has the highest number of malaria cases in China and is ranked second with regard to incidence of the disease. A spatio-temporal analysis of the epidemic situation from 1999–2005 in Yunnan found significant clustering. The researchers found that the lowest incidence rate of malaria due to *Plasmodium falciparum *occurred in 2000 and that of *P. vivax *malaria in 2001. The highest incidence rate of malaria in general occurred in 2003 when it increased considerably, particularly malaria due to *P. falciparum *which has increased by more than 100%. The incidence has fluctuated during the past seven years but the proportions of *P. vivax *malaria and *P. falciparum *malaria held steady at 73% and 22%, respectively. The malaria season mainly occurs from May to August and from October to November. The fastigium is in July and November and the low point in September and February. The first fastigium for *P. falciparum *malaria is in June, which is one month earlier than for *P. vivax *malaria.

In China, the Huaihe River valley is an endemic area with substantial increase in recent years. The source of the infection and the vector are particularly difficult to control due to various reasons and social factors related to the epidemic [118]. Zhigui Xia found that the ratio of using door-window screen, bednet and repellent sprays (β = -2.13, -1.15, -1.03, respectively, P < 0.05) had a negative correlation to the natural logarithm rate (LnY), while the rate of sleeping or chatting outdoors (β = 0.89, P < 0.01) had positive LnY correlation.

#### Lymphatic filariasis

Lymphatic filariasis remains an important parasitic disease in some countries in Southeast Asia [119] even if it has recently been deemed eliminated in China [120]. Although most of Cambodia is free from the disease, Lek Dysoley reported that filariasis is still prevalent in four northern provinces (Rattana Kiri, Stung Trenga, Preah Vihear, Siem Reap) [121].

In Indonesia, on the other hand, a survey conducted in 2005 showed the disease is widespread and epidemic. A study of 1,250 individuals from seven villages of Banggai district and 437 individuals from two villages of Parigi-Moutong district examined using finger prick night blood survey, showed filariasis to be endemic in three villages. Two carriers of *Wuchereria bancrofti *microfilaria were detected in two of them (Dongin and Bone Bae) on the coastal area of the Banggai district. Microfilaria of *Brugia malayi *was found in 28 individuals in one village (Pangku-Tolole) of the Parigi-Moutong district and the geometric mean of microfilarial density was 6.97 mf/mm^3 ^and 23.71 mf/20 mm^3 ^in male and female, respectively. The highest densities of microfilaria were observed in the 10–39 year olds. This notwithstanding, the highest mf density (181 mf/20 mm^3^) was found in a 50-year-old woman. *B. malayi *in the Parigi-Moutong district was found to be nocturnally periodic in principle, but a small number of mf were also detected in the day blood of two of five mf carriers who were examined for periodicity [122,123].

An investigation on vector distribution and biology, conducted in Pangku-Tolole village, Parigi-Moutong districts, province of central Sulawesi, Indonesia, collected *An. barbirostris*, *An. flavirostris*, *An. vagus*, *Ae. vexans*, *Culex vishnui*, *Cx. gelidus*, *Cx. bitaeniorhynchus *and *Mansonia uniformis*, but only *An. barbirostris *was found to be infected with an infection rate of approximately 10% [124]. Although the microfilaria prevalence was 13.5% in this village with 207 residents, 37.5% reported a history of acute filarial attacks and two individuals presented with severe elephantiasis. More than 10% of the 123 households in Pangku-Tolole were included in a risk factors survey. The result showed that poor knowledge and outdoor activity at night were associated with filarial infection. Additionally, the environmental condition and poor sanitary facilities in this area facilitates vectors to breed [125].

## Conclusion

The 1^st ^International Symposium on Geospatial Health presents new evidence that spatially-explicit analyses and other geospatial approaches with an emphasis on human health are already widely applied across Southeast Asia. Indeed, in some cases, tools and methodologies have been further developed and refined on site for specific purposes. The key findings have been summarized here not only to emphasize advances made but also to identify current research priorities. Applications presented and discussed at this symposium range from soil-transmitted to food-borne and water-borne helminthic infections in addition to lymphatic filariasis, leishmaniasis and malaria. As a matter of course, the seriousness of these infections varies from country to country but, taken together, their impact in Southeast Asia is substantial, yet seriously underestimated and often neglected.

Technical progress in the field of GIS and RS techniques, including geospatial statistics, has provided a new epidemiological approach which is particularly well suited to the study of climate change and ecological transformations. For example, time series analyses, advanced modeling and Bayesian geostatistics are widely employed to deepen our understanding of the epidemiology and control of schistosomiasis, including the use of random-effect models and transmission dynamics. Convincing data were presented showing that schistosomiasis may progress further north in a warmer future China. For these kinds of investigations, in particular, the geospatial tools can provide early-warning techniques for preemptive planning in order to mitigate future risk and impact. We now have an essentially full understanding of infectious agents and their life cycles. Ambient temperatures strongly influence the distribution of the parasitic, infectious agents, the intermediate hosts and vectors involved, and their interaction. However, as underlined by many communications given during the symposium, there are also many other important, remotely-sensed variables that are highly useful to follow in order to produce fully animated illustrations how these infections wax and wane.

It is of particular interest that there is now a strong focus on monitoring the effects due to large-scale ecological and demographic transformations in connection with the Three Gorges Dam completed in 2009, and the South-to-North Water Transfer Project under construction currently. China clearly needs the provision of electricity and water, but it must also deal with the potentially far-reaching consequences, e.g. epidemic spread of certain diseases, which may well accompany these large-scale water resources development and management projects. Work presented at the symposium supports the notion that the progress of remotely-sensed environmental data and advanced mathematical modeling in a spatially-explicit framework is making it possible to keep abreast of the demographic, eco-epidemiological and socio-economic changes that may arise and also highlights the need to develop early-warning systems without delay.

Many investigations in the areas of geostatistics, visualization and modelling of disease transmission were presented in the form of mathematical models based on multi-disciplinary principles. In order to efficiently improve the accuracy of prediction and simulation under a host of different scenarios, assessment of prediction and models should be explored further to enhance our understanding of control strategies and rigorous monitoring of interventions. It is recommended that research priorities within the field of geospatial health focus on the development of advanced spatial analytical tools to promote their application in human public health and veterinary medicine.

## Competing interests

The authors declare that they have no competing interests.

## Authors' contributions

XNZ and SL wrote the first version of the manuscript. GJY, TKK, NRB, JU, and JBM revised the manuscript. XNZ, NRB, and JU finalized the manuscript. All of authors read and approved the final version of the manuscript.
